# Adolescent friendships predict later resilient functioning across psychosocial domains in a healthy community cohort

**DOI:** 10.1017/S0033291717000836

**Published:** 2017-04-11

**Authors:** A.-L. van Harmelen, R. A. Kievit, K. Ioannidis, S. Neufeld, P. B. Jones, E. Bullmore, R. Dolan, The NSPN Consortium, P. Fonagy, I. Goodyer

**Affiliations:** 1 Department of Psychiatry, University of Cambridge, Cambridge, UK; 2 Medical Research Council, Cognition and Brain Sciences Unit, Cambridge, UK; 3 Wellcome trust Center for Neuroimaging, University College London, London, UK; 4 Department of Clinical, Educational and Health Psychology, University College London, London, UK

**Keywords:** Adolescence, Friendships, Child Adversity, Resilience, Mental health

## Abstract

**Background:**

Adolescence is a key time period for the emergence of psychosocial and mental health difficulties. To promote adolescent adaptive (‘resilient’) psychosocial functioning (PSF), appropriate conceptualisation and quantification of such functioning and its predictors is a crucial first step. Here, we quantify resilient functioning as the degree to which an individual functions better or worse than expected given their self-reported childhood family experiences, and relate this to adolescent family and friendship support.

**Method:**

We used Principal Component and regression analyses to investigate the relationship between childhood family experiences and PSF (psychiatric symptomatology, personality traits and mental wellbeing) in healthy adolescents (the Neuroscience in Psychiatry Network; *N* = 2389; ages 14–24). Residuals from the relation between childhood family experiences and PSF reflect resilient functioning; the degree to which an individual is functioning better, or worse, than expected given their childhood family experiences. Next, we relate family and friendship support with resilient functioning both cross-sectionally and 1 year later.

**Results:**

Friendship and family support were positive predictors of immediate resilient PSF, with friendship support being the strongest predictor. However, whereas friendship support was a significant positive predictor of *later* resilient functioning, *family* support had a *negative* relationship with later resilient PSF.

**Conclusions:**

We show that friendship support, but not family support, is an important positive predictor of both immediate and later resilient PSF in adolescence and early adulthood. Interventions that promote the skills needed to acquire and sustain adolescent friendships may be crucial in increasing adolescent resilient PSF.

## Introduction

Adolescence is a key developmental time period for the emergence of psychosocial difficulties, and mental health disorders (Thapar *et al.* 2012; Blakemore & Mills, 2014). To promote adaptive psychosocial functioning (PSF) during adolescence, appropriate conceptualisation and quantification of resilient functioning and its predictors is a crucial first step. In psychiatry, resilience refers to “*a dynamic process wherein individuals display positive adaptation despite experiences of significant adversity or trauma*” (Luthar & Cicchetti, 2000). In the general population, it is well established that negative childhood experiences such as parental discord and/or lack of parental affection can have a negative impact on adolescent PSF (Egeland, 2009; Trocmé *et al.* 2011; van Harmelen, 2013; Harpur *et al.* 2015; Stoltenborgh *et al.* 2015). Adolescent resilient PSF may therefore be seen as reflecting positive adaptation compared to others with similar experiences in the family environment. However, individuals with comparable experiences may not *appraise* their experiences in the same way (Rutter, 1985, 2012). For instance, *perceived* levels of threat, rather than actual threat, determine later stress reactivity (van Wingen *et al.* 2011). Therefore, including self-reported appraisal of childhood family experiences may contribute to a more valid and quantifiable measure of adolescent resilient functioning.

Resilience captures positive adaptation across emotional, cognitive, behavioural and social domains of functioning (Masten, 2015), and should be relevant to the environmental events and difficulties experienced (Luthar *et al.* 2000). From this multidimensional perspective the presence of personal impairment or psychopathology does not necessarily preclude concurrent resilient functioning (Luthar *et al.* 2000). For example, an adolescent can suffer considerable distress after a personal loss, but simultaneously continue to attend school and learn and see friends and can therefore be considered to be functioning ‘resiliently’ in those domains despite experiencing berievement. This multidimensional perspective indicates that a valid measure of adolescent resilient functioning in the general population should capture adaptive behaviour across a comprehensive range and level of psychosocial domains. Furthermore, resilient functioning is not a personality trait that is constant over time (Luthar & Cicchetti, 2000; Rutter, 2012; Cicchetti, 2013; Masten, 2015). Rather, resilient functioning waxes and wanes, possibly under the influence of protective factors such as family and friendship support (Rutter, 1985; Afifi & Macmillan, 2011; Cicchetti, 2013; van Harmelen *et al.* 2016). Therefore, having low resilience at some time does not preclude the presence of future resilience or vice versa. Consequently, it is important to study adolescent resilient functioning, and its influences, over time (Bonanno *et al.* 2015). Understanding how adolescent resilient functioning varies over time and revealing how various factors influence such variation remains to be fully elucidated.

Adolescent friendships and family support are important protective factors after early life stress (Rutter, 1985, 2012, van Harmelen *et al.* 2016). Recently, we showed that adolescent family support reduces later depressive symptoms after differential levels of childhood family adversity, whereas adolescent friendship support reduced later depressive symptoms after childhood family adversity and/or peer victimisation (van Harmelen *et al.* 2016). These findings support the stress-support matching hypothesis; support should match the type of adversity experienced in order to be most beneficial (Cohen & Wills, 1985). This is also evidence for multidimensionality of resilient functioning and further evidences the value of developing a resilient index across domains of experiences. Such a measure has however yet to be reported. Thus, the concurrent and predictive role of adolescent friendships and family supports on adolescent psychosocial resilient functioning across multiple domains (whilst taking self-reported childhood family experiences into account) is unknown.

Here, we investigate the relationship between adolescent family and friendship support on concurrent and prospective adolescent resilient PSF in a community sample (*N* = 2389) of healthy adolescents and young adults (ages 14–24) from the longitudinal Neuroscience in Psychiatry Network (NSPN; http://www.NSPN.org). We quantify a measure of resilient functioning by taking into account both functioning across multiple psychosocial domains (i.e. psychiatric symptoms, personality traits and mental wellbeing) and self-reported experiences of the family environment in childhood in a healthy population. This allows us to create a multidimensional index of functioning from which we can ascertain the degree to which an individual functions better or worse than expected given their family environment in early life. Finally, we test whether such functioning is associated specifically with family and friendship factors concurrently and prospectively 1 year later using path models.

## Methods

### Sample

Participants in this report were part of the NSPN study cohort. NSPN is a multi-centre accelerated longitudinal community cohort study focusing on normative adolescent to young adult (‘adolescent’) development between the ages of 14 and 24. The NSPN cohort (*N* = 2389) completed a home questionnaire pack (HPQ) at baseline (Time 1), and ~1 year later [median = 1 year, mean = 1.11 (s.e. = 0.01) year, min–max: 0.91–2.69 years], *N* = 1674 individuals from the NSPN cohort completed the same HPQ at Time 2.

For our cross-sectional analyses we had complete data on all measures used (online Supplementary Table S1) for *N* = 1890. This cross-sectional sample did not differ from the entire NSPN cohort (*N* = 2389) on age [*t* (4055) = 0.02, *p* = 0.98], gender (χ^2^ = 0.01, df = 1, *p* = 0.91), socio-economic status [SES; index of multiple deprivation based on participant postcodes; *t* (4058) = 1.416, *p* = 0.16], nor ethnicity (χ^2^ = 4.19, df = 5, *p* = 0.52). Overall, online Supplementary Table S1 shows that this sample (*N* = 1890) can be described as a healthy sample reporting low levels of psychopathological symptoms, behaviours and personality traits, and average mental wellbeing scores.

For our longitudinal analyses we had complete data for *N* = 1093. This longitudinal sample was not different from the sample used in our cross-sectional analyses (*N* = 1890), nor the entire NSPN cohort (*N* = 2389) in terms of age [*t* (2058 & 2218)< −0.74, *p* > 0.46], and ethnicity distribution [i.e. *N* = 1890: (χ^2^ = 2.73, df = 5, *p* = 0.74); *N* = 2389: (χ^2^ = 9.15, df = 5, *p* = 0.10)]. However, there were slightly more females in the longitudinal sample (*N* = 1093; 57% females) when compared with the cross-sectional sample (*N* = 1890) and the NSPN cohort (*N* = 2389) (χ^2^ > 4.16, df = 1, *p* < 0.04), that both had 53% females. Finally, the longitudinal sample (*N* = 1093) had similar SES compared with the cross-sectional sample (*N* = 1890) *t* (2364) = −1.5, *p* = 0.13). However, the longitudinal sample (*N* = 1093) had lower SES compared with the NSPN cohort (*N* = 2389) (mean's 15.5 & 16.9, *t* (2237) = −2.82, *p* = 0.005).

### Measures

#### Psychosocial functioning (PSF)

Negative family environments in early life form a risk factor for maladaptive-psychiatric symptomatology (van Harmelen *et al.* 2010), -personality traits (Hart *et al.* 1997; Rogosch & Cicchetti, 2004) and reduced overall mental wellbeing (Hart *et al.* 1997). Therefore, we focussed our measure of resilient functioning relative to these psychosocial domains to assess overall ‘*PSF*‘†^1^. To do so, we included sum scores of all questionnaires (assessed both at times 1 and 2) that focussed on:

Psychopathological symptoms: The mood and feelings questionnaire (Angold *et al.* 1995), Revised Children's Manifest Anxiety Scale RCMAS self-report questionnaire (Reynolds & Richmond, 1997), Short Leyton Obsessional Inventory (Bamber *et al.* 2002), Kessler Psychological Distress scale (K10; Kessler *et al.* 2002), behaviours checklist.

Personality characteristics: The Antisocial Process Screening Device (Frick *et al.* 2000), The Child and Adolescent Dispositions Scale (Lahey *et al.* 2008), the inventory of Callous-unemotional traits (ICU) to measure callous and unemotional traits (Roose *et al.* 2010), the Schizotypal Personality Questionnaire (SPQ) (Raine, 1991) and the Barratt Impulsivity Scale (BIS) (Stanford *et al.* 2009).

Mental wellbeing: the Warwick-Edinburgh Mental Well Being Scale (WEMWBS) (Tennant *et al.* 2007).

More information about these measures is provided in the online Supplement.

#### Childhood family experiences

Appraisal of early life parenting behaviours were measured at baseline and time 2 with two self-report measures; the Alabama Parenting Questionnaire (APQ) and the measure of parenting styles (MOPS).

*Measure of Parenting Style*: The MOPS is a 12-item self-report measure that assesses perceived parenting styles across three domains; indifference, overcontrol and abuse (Parker *et al.* 1997). Participants were asked to rate both their mother's and father's parenting behaviour on 15 statements, on a 4-point scale. The full response range is ‘not true at all’, ‘slightly true’, ‘moderately true’, ‘extremely true’. The ‘abuse’ scale consisted of five items, asking whether maternal/paternal behaviours were verbally abusive, unpredictable, physically violent, elicited feelings of danger or elicited feelings of lack of safety. The ‘overly controlling’ scale consisted of four items where maternal/paternal behaviour was overprotective, over controlling, critical, or made the participant feel guilty. Finally, the ‘indifference’ scale assessed six items of maternal/paternal behaviour where the parent was ‘ignoring, uncaring, rejecting, uninterested in, would forget about, or would leave the participant on his/her own a lot. Sum scores to responses in these items were calculated with higher scores representing more abusive, over controlling or indifferent behaviour reported. Internal consistency was good for the maternal subscales (Cronbach's alpha maternal over control = 0.70, indifference = 0.86, abuse = 0.78). For paternal parenting, the internal consistency at baseline ranged from acceptable (Cronbach's alpha paternal over control = 0.65) to excellent (Cronbach's alphas paternal abuse = 0.88, paternal indifference = 0.93).

*Alabama Parenting Questionnaire*: The APQ measures parenting practices. We used the nine-item short-form (Elgar *et al.* 2006), and added the ‘Corporal Punishment’ (three items) and ‘Involvement’ scale (three items). Participants were asked to rate how typical each item occurred or used to occur in their family home on a 5-point scale ranging from ‘*never*’, ‘*almost never*’, ‘*sometimes*’, ‘*often*’ *to* ‘*always*’. We calculated sum scores for the five subscales: Positive Parenting, Inconsistent Discipline, Poor Supervision, Involvement, and Corporal Punishment, with higher scores reflecting higher frequency of the behaviour. Thus, high scores can indicate positive parenting (i.e. involvement, positive parenting) or negative parenting (i.e. inconsistent discipline, poor supervision, corporal punishment). Internal consistency at baseline was acceptable (inconsistent discipline & poor supervision: Cronbach's alpha > 0.62) and good (positive parenting, involvement, Corporal Punishment Cronbach's alpha > 0.71). Note that all results remained when the positive parenting scores (APQ positive parenting and APQ involvement) were removed from the analyses.

#### Predictors of resilient functioning

*Family Assessment Device (FAD)*:

Adolescent family support was assessed at baseline and time 2 with the McMaster FAD-General Functioning Scale (FAD-GF; Epstein *et al.* 1983), administered to adolescents. The FAD-GF is a 12-item self-report questionnaire where respondents rate statements such as ‘we can express our feelings to each other’ or ‘there are lots of bad feelings in the family’. Responses ranged from ‘Strongly Agree’ to ‘Strongly Disagree’. The FAD-GF yields an estimate of overall family functioning (Miller *et al.* 1985). In our analyses, high scores reflect a positive family environment (‘family support’). Internal consistency at baseline was very high (Cronbach's alpha = 0.92).

*Cambridge Friendship Questionnaire (CFQ)*: Perceived quality of friendships at baseline and time 2 were assessed with the self-report CFQ (J. Memarzia *et al.* unpublished observations; van Harmelen *et al.* 2016). The CFQ is an eight-item questionnaire assessing the number, availability and quality of friendships (e.g. ‘Do you feel that your friends understand you?’, ‘Are you happy with the number of friends that you've got at the moment’, ‘Can you confide in your friends?’). Higher scores indicate better perceived overall quality of friendships (i.e. ‘Friendships’). The CFQ has good measurement invariance and external validity, and adequate test–retest reliability across 2-week intervals (Kappa = 0.80) (J. Memarzia *et al.* unpublished observations). Within NSPN, baseline internal consistency was good (Cronbach's alpha = 0.72).

### Stats and results

All analyses were conducted in R version 3.03 (Warm Puppy), using the packages Dplyr (Wickham & Romain, 2016), Psych (Revelle, 2014), Lavaan (Rosseel, 2012) and ggplot2 (Wickham, 2009). All data and code for the below analyses are available from (http://www.annelauravanharmelen.com/data & https://figshare.com/authors/_/1376682).

To calculate a multi-modal composite score for PSF we conducted a principal component analysis (PCA) for PSF on standard-normally transformed individual total scores on the MFQ, RCMAS, S-LOI, K10, BCL, APSD, CADS, ICU, SPQ, BIS-11 and WEMBES. Similarly, we conducted a PCA, including standard-normally transformed sum scores for the MOPS the APQ subscales to create a composite score for childhood family experiences. From both analyses, we extracted individual scores for the first component to reflect individual current PSF and recalled childhood family experience scores. Next, we regressed the PSF component score against the childhood family experiences score, testing for possible linear, quadratic or cubic relationships. From the best-fitting regression we extracted the residual scores as these reflect a spectrum ranging from *risk to resilient* functioning: *the extent to which an individual has better, or worse, PSF outcomes than the average score expected given their childhood family experiences* (see for a similar approach Bowes *et al.* 2010; Miller-Lewis *et al.* 2013; Sapouna & Wolke, 2013; Collishaw *et al.* 2016). For parsimony, we will refer to these scores as ‘*resilient functioning*’ with higher scores reflecting better (conditional) PSF outcomes.

Next, we predicted resilient functioning from adolescent family and friendship support. Age, gender (coded 0-1, 1 being males) and socio-economic status (SES) were specified as covariates. Note that all results remained the same when these covariates were not included in the regressions. We examined these relations cross-sectionally at baseline in *N* = 1890 using multiple regression.

Finally, we investigated whether the relationships between friendship and family support and our multidimensional measure of resilient functioning is dependent on the cross-sectional (i.e. simultaneous) timing of assessments, (potentially reflecting reporting bias) or whether these relationships also appeared over time. Therefore, we conducted longitudinal analyses using Structural Equation Modelling (SEM) in Lavaan (Rosseel, 2012). We specified a full identified model that tested the relations and interrelations of baseline and later friendships, family support and resilient functioning. In this model, gender, age and SES were specified as covariates on friendships, family support and resilient functioning at baseline and follow-up.

## Results

### Resilient functioning; functioning that is better than expected given one's childhood family experiences

**Fig. 1.**
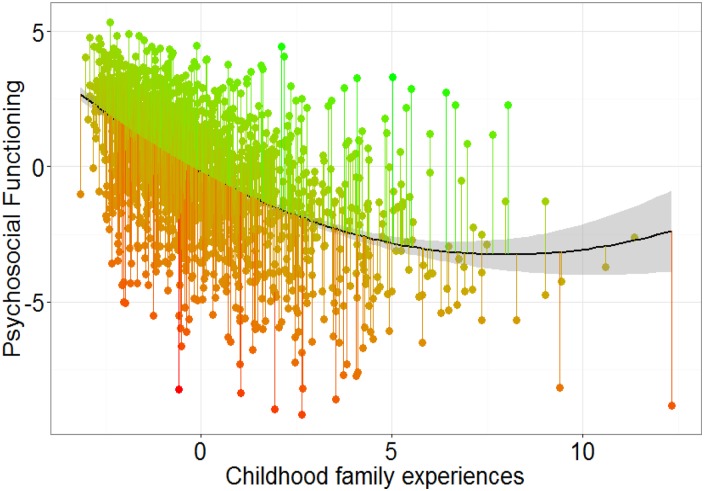
Relationship between PSF and Childhood family experiences in *N* = 1890.

A PCA for PSF (MFQ, RCMAS, S-LOI, K10, BCL, APSD, CADS, ICU, SPQ, BIS-11 and WEMBES) revealed a first component that explained 44% variance. Higher scores on the PSF factor suggest better PSF (see online Supplementary Table S2). The PCA for child family experiences revealed a first component that explained 37% variance in the MOPS and APQ subscales (online Supplementary Table S2 for loadings). The childhood family experiences principle component scores were inverted so that a higher score reflects more negative family experiences. We next regressed the childhood family experiences component score on the component score for PSF. This relationship could best be described as quadratic (Fig. 1) (Est = −0.76, s.e. = 0.03, *t* = −24.87, *p* < 2 × 10^−16^, quadratric term: Est = 0.05, s.e. = 0.006, *t* = 7.32, *p* = 3.66 × 10^−13^, additional information in online Supplement). Next, individual residual scores were extracted from this relationship as these residuals reflect degree of risk to resilient functioning: *the extent to which an individual functioned better than expected* (‘*high, or resilient*’; *green lines* Fig. 1), *or worse than expected* (‘*low or risk*’ *red lines* Fig. 1), *given their childhood family experiences.* Note that higher residual scores reflect *more* resilient functioning.

### Association between adolescent friendships and family support and resilient functioning

**Fig. 2.**
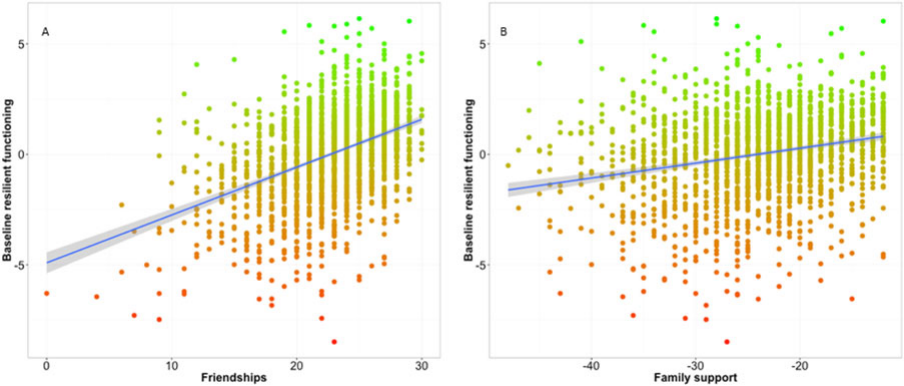
The relationship between friendships (*a*) and family support (*b*) and baseline resilient functioning (*N* = 1890).

Adolescent friendships had a strong positive association with concurrent resilient functioning; more friendship support related to more resilient functioning [*r* = 0.43, *t* (1834) = 20.57, *p* < 2.2 × 10^−16^, Fig. 2*a*]. Similarly, family support was positively associated with concurrent resilient functioning [*r* = 0.23, *t* (1853) = 10.37, *p* < 2.2 × 10^−16^, Fig. 2*b*].

Friendships and family support were correlated ]*r* = 0.39, *t* (1834) = 18.67, *p* < 2.2 × 10^−16^]. Therefore, we next investigated their unique relations with resilient functioning using multiple regression. We defined friendships, family support, gender, age and SES as predictors of resilient functioning. This analysis showed that friendships and family support were both positive predictors of resilient scores, with friendships being the strongest predictor (Table 1). Furthermore, age and male gender, but not SES, were also associated with resilient functioning.

**Table 1.** Predictors of resilient functioning at baseline (time 1)

Baseline		Estimate	β	s.e.	*t*	*p* (>|*t*|)	
Entire sample	Friendship	0.21	0.41	0.01	17.90	<2 × 10^−16^	***
(*N* = 1890)	Family	0.02	0.06	0.01	2.81	0.00	**
	Age	0.05	0.07	0.01	3.22	0.00	**
	Gender	0.31	0.08	0.08	3.67	0.00	***
	SES	0.00	−0.01	0.00	−0.40	0.69	

### Longitudinal predictors of resilient PSF

To investigate the relationship between friendships and family support at baseline (time 1) with resilient functioning at time 2 (~1 year later) we recalculated resilient functioning scores in a subset of the sample that had complete data on all measures at both times (*N* = 1093; see online Supplement and Supplementary Table S4 for details). Resilient functioning at times 1 and 2 had a strong positive association (*r* = 0.66, *t* = 28.93, df = 1091, *p* < 2.2 × 10^−16^), suggesting that resilient functioning is relatively stable over the course of 1 year in our sample.

**Fig. 3.**
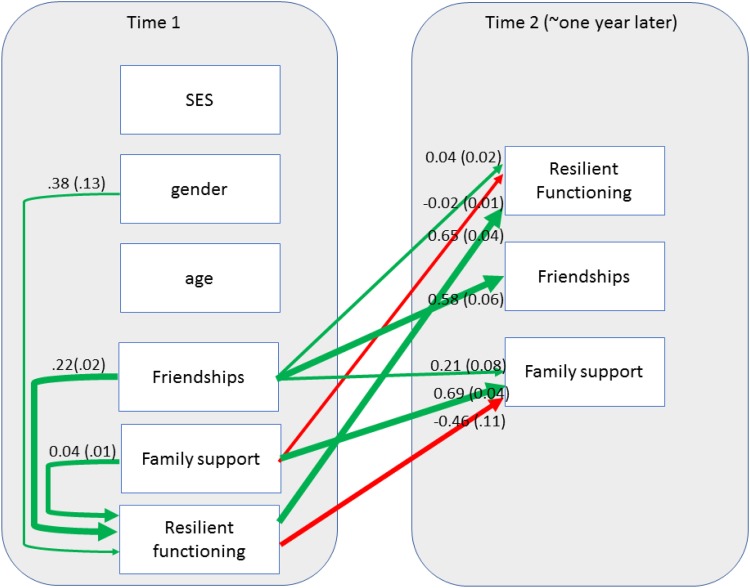
Significant paths in the Structural Equation Model. For reasons of parsimony we only depict significant positive (green) or negative (red) paths (unstandardised Estimates and s.e.). Thicker lines indicate stronger associations.

A path analysis showed that adolescent friendships and resilient functioning were significant *positive* predictors of psychosocial resilient functioning over the course of 1 year (Table 2 and Fig. 3). In contrast, adolescent family support was *negatively* associated with later psychosocial resilient functioning. Friendships at time 2 were only positively predicted by baseline resilient functioning; whereas family support at time 2 was positively predicted by baseline family *and* friendship support. Interestingly, family support at time 2 was negatively predicted by baseline resilient functioning. Note that as this path model is saturated, model fit is not informative, but included in the caption of Table 2 for completeness.

**Table 2.** Predictors of later resilient functioning (N = 1093)

Dependent variable	Predictors	Estimate	s.e.	*z*-value	*p* (>|*z*|)
Resilient functioning time 1	Friendships time 1	0.219	0.023	9.467	0.000
	Family time 1	0.036	0.01	3.494	0.000
	Age time 1	0.03	0.021	1.459	0.144
	Sex	0.378	0.127	2.98	0.003
	SES	0.001	0.005	0.143	0.887
Resilient functioning time 2	Resilient functioning time 1	0.653	0.035	18.563	0.000
	Friendships time 1	0.041	0.017	2.435	0.015
	Family time 1	−0.02	0.008	−2.422	0.015
	Age time 1	0.019	0.016	1.232	0.218
	Sex	0.041	0.1	0.409	0.682
	SES	0.005	0.004	1.286	0.199
Family time 2	Resilient functioning time 1	−0.459	0.114	−4.033	0.000
	Friendships time 1	0.205	0.081	2.541	0.011
	Family time 1	0.69	0.042	16.516	0.000
	Age time 1	0.019	0.072	0.269	0.788
	Sex	−0.039	0.451	−0.085	0.932
	SES	−0.016	0.018	−0.865	0.387
Friendships time 2	Resilient functioning time 1	0.136	0.077	1.763	0.078
	Friendships time 1	0.581	0.059	9.788	0.000
	Family time 1	0.006	0.021	0.274	0.784
	Age time 1	−0.046	0.042	−1.095	0.274
	Sex	−0.14	0.25	−0.56	0.576
	SES	−0.007	0.009	−0.84	0.401
modelfit	χ^2^(0) = 0, *p* = NA, CFI = 1, TLI = 1, RMSEA = 0(0–0)				

## Discussion

Here we examine predictors of adolescent resilient functioning across a range of psychosocial domains (‘PSF’; i.e. psychiatric symptoms, personality traits and mental wellbeing), whilst taking into account individual childhood family experiences. We create a measure of resilient PSF in three steps. First, we use a data-reduction technique (i.e. PCA) to establish individual composite scores for PSF, and childhood family experiences. Second, we regressed PSF on childhood family experiences. Third, we extract residual scores from this relationship as these reflect individual level of psychosocial resilient functioning: *the degree to which a participant is functioning better or worse than expected based on his/her childhood family experiences:* see for a similar approach (Bowes *et al.* 2010; Miller-Lewis *et al.* 2013; Sapouna & Wolke, 2013; Collishaw *et al.* 2016). We found that childhood family experiences have a significant association with PSF in our community sample of healthy adolescents (*N* = 1890). Specifically, recalling more negative family experiences was associated with worsening current PSF, supporting previous studies (Gilbert *et al.* 2009; van Harmelen *et al.* 2010). We then related adolescent friendship and family support with continuous risk to resilient PSF measure. We found that adolescent friendship support, but not adolescent family support, was positively related with immediate *and* later resilient PSF.

Friendship and family support were both positive predictors of *immediate* resilient PSF. Notably, friendship support was a stronger predictor of immediate resilient functioning than family support, which is in line with the notion that adolescents are especially sensitive to their peer environment (Crone & Dahl, 2012). Furthermore, we found that adolescent friendship support was also a positive predictor of *later* resilient PSF, which was apparent even after accounting for the effect of baseline resilient functioning and family support. These findings suggest that friendship support may be an important protective factor in adolescence. Our findings corroborate and extend those that showed that adolescent friendship support promotes subsequent resilient functioning in those exposed to negative childhood family environments (Collishaw *et al.* 2007; Powers *et al.* 2009; van Harmelen *et al.* 2016).

The exact mechanisms through which adolescent friendships increases resilient functioning are yet unknown. One potential explanation may be that our friendships score captures individual skills that promote social competence, such as social interaction and relationship building, and social competence could mediate the link between resilient functioning and friendship interactions. However, in our model, the relationship between baseline resilient functioning and later friendships was weak at best. This suggests that our interpretation that friendship promote resilient functioning over time is unlikely to be explained by the alternative notion that prior resilient functioning promotes better social competence (and friendships) and thereby later resilience function. Future studies should however test the specific role of social competence in the link between resilient functioning and subsequent friendships. Other explanations for the link between friendships and resilient functioning may come from studies that suggest that adolescent friendship support may increase resilient functioning through offering companionship (Cohen & Wills, 1985) when these interactions are pro-social, as adolescent prosocial peer relationships, but not anti-social relationships, reduced later behavioural problems (Fergusson & Lynskey, 1996; Fergusson *et al.* 1996). Friendships may also increase resilient functioning is through increasing interpersonal skills (Buhrmester, 1990), and through supporting social decision making skills (Jehn & Shah, 1997). Additionally, adolescent friendships may reduce feelings of loneliness (Parker & Asher, 1993), and dampen stress responses (Cohen & Wills, 1985; Masten *et al.* 2012). Furthermore, friendship support may increase resilient functioning through reducing negative experiences with peers (Pellegrini & Bartini, 2000).

Overall therefore these emotion–cognition mechanisms accruing via positive adolescent friendships may increase resilient functioning through updating negative self-cognitions. Negative self-cognitions are found in children that have low peer support; those that have experienced peer victimisation (Sinclair *et al.* 2012), or report to be lonely (Vanhalst *et al.* 2015). Negative self-cognitions colour individuals' appraisal and behaviour in interpersonal situations and negatively influence individuals' memories of these situations (Beck, 2008). Negative self-cognitions mediate the link between very negative family environments and poor mental health (van Harmelen *et al.* 2010). Adolescent friendship support may offer a unique opportunity to learn from positive peer experiences, which perhaps results in a more positive update of self-cognitions. Examining the potential mechanisms through which adolescent friendship support increases psychosocial resilient functioning is an important avenue for future research.

The relationship between adolescent *family* support and resilient functioning across psychosocial domains appeared to be more complicated in our sample. Although family support had a *positive* relationship with immediate resilient PSF, family support was *negatively* related with *later* adolescent resilient functioning (when baseline resilient functioning and friendship support were taken into account). These findings are in line with findings that family support is not linked to positive adaptation in more severely maltreated children than those studied here (Cicchetti, 2013). It may be that, in adolescence, family involvement is not adaptive, especially in the context of a negative family environment. In line with this idea, adolescent family support was not associated resilient functioning when peer relationships were taken into account (Fergusson & Lynskey, 1996). Similarly, family support was not associated with teacher-reported mental health resilient functioning in young children with parental report of high cumulative family adversity (Miller-Lewis *et al.* 2013). Although, family support was positively related with mental health resilient functioning if functioning was reported by parents in these children (Miller-Lewis *et al.* 2013). Finally, our findings are in line with those that friendships, but not family support, are related with self-reported resilient functioning rates on a resilient functioning questionnaire in young adults with histories of child abuse (Howell & Miller-Graff, 2014). However, our findings are in contrast with those that suggest that family support is predictive of childhood and early adolescent (ages 13–14) resilient functioning against depressive symptoms after child adversity (Bowes *et al.* 2010; Sapouna & Wolke, 2013). These findings also contrast our previous report in a different sample that adolescent family support at age 14 reduces adolescent depressive symptoms at age 17 after CFA (van Harmelen *et al.* 2016). Taken together, whereas previous studies suggest that early adolescent family support may predict resilient functioning against later depression, our current findings suggest that adolescent family support is not related with later adolescent resilient functioning when resilient functioning is assessed across multiple psychosocial domains.

Contrary to common concepts of resilient functioning where only the outcomes (e.g. absence of psychopathology, above average functioning) are taken into account [e.g., see for an overview (Klika & Herrenkohl, 2013)], we use an approach that allows individuals who have moderate outcomes in the face of very negative childhood family experiences to be included as ‘resilient’ (Bowes *et al.* 2013; Miller-Lewis *et al.* 2013; Sapouna & Wolke, 2013; Collishaw *et al.* 2016). This approach paints a more complete picture of adolescent PSF. A limitation of this approach is that taking the subjectivity of self-reported childhood family experiences into account when quantifying resilient functioning may be inherently biased: those that are highly resilient may report more positive childhood family experiences, whereas those that are less resilient may report more negative childhood family experiences. However, current psychopathology has not been found to bias self-report of child abuse and neglect (Spinhoven *et al.* 2010). In fact, a previous work suggests that negative childhood experiences are more likely to be *underreported* rather than overreported (Brewin, 2007). Finally, even if those with low psychosocial resilient functioning overreported negative family experiences, and those with high psychosocial resilient functioning over reported positive family experiences this would only lead to a *reduction* in power to find associations with resilient functioning. For these reasons, it is unlikely that this limitation would explain our current findings. Finally, an important limitation is that, on average, our sample reported only low levels of negative family experiences at best, and the childhood family experiences score explained only moderate variance *r* = 0.37% in the MOPS and APQ assessments. Future studies should investigate whether friendship support similarly predicts resilient PSF after more severe childhood family experiences including a sufficient sample of adolescents with manifest histories of physical and sexual maltreatment in childhood that are not studied in this investigation.

In sum, we quantify resilient functioning by taking into account functioning across a range of psychosocial domains *and* individual childhood family experiences. We show that friendship support, but not family support, is an important positive predictor of both immediate and later resilient PSF in adolescence and early adulthood. Therefore, interventions that promote the skills needed to acquire and sustain adolescent affiliate friendships may be crucial in increasing adolescent resilient functioning.

## Footnotes

† The notes appear after the main text.

## Note

1 As our aim is to capture resilient functioning in its broadest sense, we choose to incorporate the more stable personality traits in our measure of PSF. Note that our results remain the same when we repeated all analyses, whilst only including psychiatric symptoms and mental wellbeing in our PSF variable.

## Supplementary material

For supplementary material accompanying this paper visit http://dx.doi.org/10.1017/S0033291717000836. click here to view supplementary material
