# Supplementary material for: Adolescent friendships predict later resilient functioning across psychosocial domains in a healthy community cohort
Source: Psychol Med. 2017 Apr 11;47(13):2312–22. doi: 10.1017/S0033291717000836 (PMC5820532; doi:10.1017/S0033291717000836)
Supplement: Supplementary file 1 [file S0033291717000836sup001.docx]

Supplemental material.

Friendships predict resilient psychosocial functioning across multiple domains in a healthy community cohort of adolescents

Anne-Laura van Harmelen^1^*, Rogier A Kievit^23^, Konstantinos Ioannidis^1^, Sharon Neufeld^1^, Peter B. Jones^1^, Ed Bullmore^1^, Ray Dolan^3^, the NSPN consortium^13^, Peter Fonagy^4^ & Ian Goodyer^1^.

Contents

[Measures used to assess psychosocial functioning: 2](#_Toc472687684)

[Psychopathology symptoms: 2](#_Toc472687685)

[Personality traits: 2](#_Toc472687686)

[Mental Well Being 3](#_Toc472687687)

[Table S1, Sample descriptives. 4](#_Toc472687688)

[Table S2, Results principle component analyses for PSF and Childhood family experiences 5](#_Toc472687689)

[Regressions between relationship childhood family experiences and PSF 5](#_Toc472687690)

[Table S3 predictors of resilient functioning as calculated from the cubic relationship between PSF and childhood family experiences. 6](#_Toc472687691)

[PCA for longitudinal analysis sample (N=1093) 6](#_Toc472687692)

[Table S4, First components for PSF and childhood family experiences in N=1093. 7](#_Toc472687693)

[Figure S1. The Cubic relationship between PSF and Childhood family experiences 8](#_Toc472687694)

[Neuroscience in Psychiatry Network (NSPN) Consortium author list 9](#_Toc472687695)

## Measures used to assess psychosocial functioning:

Psychopathology symptoms:

**MFQ (Mood and Feelings Questionnaire)**

The mood and feelings questionnaire(Angold *et al.* 1995) is a 33 item self-report questionnaire measuring current (past two weeks) depressive symptoms. As only 5 of the 33 items had an endorsement of the “always” category that was above 5%, we combined all “always” responses with “mostly” responses. In our sum score, higher scores indicated more severe depressive symptoms. At baseline, internal consistency of the MFQ was excellent (Cronbach’s alpha=.93).

**RCMAS (Revised Children’s Manifest Anxiety Scale)**

We assessed anxiety symptoms with the RCMAS self-report questionnaire(Reynolds & Richmond 1997). Responses ranged from either always, mostly, sometimes or never. The internal consistency for the total sumscore was excellent at baseline (alpha=.94).

**S-LOI (Short Leyton Obsessional Inventory)**

The LOI is an 11 item self-report questionnaire that measures obsessional/anxiety symptoms(Bamber *et al.* 2002). Responses ranged from ‘always’, ‘mostly’, ‘sometimes’ to ‘never’. At baseline, the internal consistency for the sum score was good (alpha= .84).

**K10 (Kessler Psychological Distress Scale)**

We assessed psychological distress with the Kessler Psychological Distress scale (K10(Kessler *et al.* 2002; Furukawa *et al.* 2003)). Responses on the K10 range from “None of the time” to “All the time” along a five point scale. Internal consistency at baseline of the measure was very high (Cronbach’s alpha=.89).

**The behaviours checklist (BCL)**

The behaviours checklist is an 11 item self-report questionnaire for symptoms of antisocial behaviour based on DSM IV conduct disorder items. Responses on these items ranged from ‘always’, ‘mostly’, ‘sometimes’ to ‘never’. Endorsements on the “mostly” category were rare, with ten of the 11 items not reaching 5% of respondents, therefore, the “always” and “mostly” categories were combined. This measure has not been previously published. Internal consistency of the measure was good at baseline (Cronbach’s alpha=.74).

Personality traits:

**APSD (Antisocial Process Screening Device)**

The Antisocial Process Screening Device is a 20 item scale measuring psychopathic traits(Frick *et al.* 2000). Responses on the APSD are “Not at all true”, “Somewhat true” and “Certainly true”. Internal consistency of the full measure was high with Cronbach’s alpha (.73 for the entire sample), which indicates adequate internal consistency at baseline.

**CADS (Child and Adolescent Dispositions Scale)**

The Child and Adolescent Dispositions Scale(Lahey *et al.* 2008) was used to measure the dispositional traits: prosociability, negative emotionality and daring. Participants were ask to rate items on how the description best describes them (answered: “not at all”, “just a little”, “Pretty much/pretty often” and “Very much/very often”). Internal consistency at baseline for the sum scores of these dimensions was good (i.e. prosociality alpha=.78, emotionality alpha=.72, and daring=.77).

**ICU (Inventory of Callous-Unemotional Traits)**

We used the Inventory of Callous-unemotional traits (ICU) to measure callous and unemotional traits(Kimonis *et al.*; Roose *et al.* 2010). Participants were asked how well a statement described them (responses given on a four point scale ranging from “Not at all true” to “Definitely true”). Internal consistency at baseline was good (Cronbach’s alpha = .82).

**SPQ (Schizotypal Personality Questionnaire)**

The Schizotypal Personality Questionnaire (SPQ) is a 74 item scale measuring schizotypal personality traits(Raine 1991). Responses on the SPQ are a yes or no endorsement. We used a total SPQ sum score where higher scores indicated more schizotypal symptoms. In NSPN, the internal consistency of the SPQ at baseline was excellent (Cronbach’s alpha = .94).

**The Barratt Impulsivity Scale (BIS-11)**

The Barratt Impulsivity Scale (BIS)(Stanford *et al.* 2009) is a 30 item scale measuring impulsivity personality traits. Participants were asked to think about how well each item describes how they act (responses range from “rarely” to “always”). We used the total sum score, where a higher score suggests more impulsivity. Internal consistency was good (Cronbach’s alpha=.82).

Mental Well Being

**The Warwick-Edinburgh Mental Well Being Scale (WEMWBS)**

Mental well-being was assessed with the Warwick-Edinburgh Mental Well Being Scale (WEMWBS)(Tennant *et al.* 2007). Participants were asked to respond to how well each statement described their experiences in the last two week (answers ranged from “none of the time” to “all of the time” on a 5 point likert scale). We used a total sum score as total WEMWBS score where higher scores indicate better mental well-being. Internal consistency for the WEMWBS was very high at baseline (Cronbach’s alpha = .92).

## Table S1, Sample descriptives.

| Time 1 (baseline) (N=1890): |  |  | Median | Mean | SD | SE | min | max |
| --- | --- | --- | --- | --- | --- | --- | --- | --- |
| PSF | MFQ total score | | 15 | 17.54 | 11.54 | 0.27 | 0 | 64 |
|  | RCMAS total score | | 16 | 17.82 | 11.99 | 0.28 | 0 | 56 |
|  | LOI total score | | 3 | 4.77 | 4.84 | 0.11 | 0 | 32 |
|  | BEH total score | | 1 | 1.19 | 1.84 | 0.04 | 0 | 20 |
|  | k10 total score | | 8 | 9.46 | 7.02 | 0.16 | 0 | 39 |
|  | APSD total score | | 11 | 11.77 | 4.42 | 0.1 | 2 | 31 |
|  | CADS prosoc | | 42 | 41.04 | 5.36 | 0.12 | 18 | 52 |
|  | CADS negemot | | 15 | 15.19 | 3.83 | 0.09 | 7 | 28 |
|  | CADS daring | | 12 | 12.22 | 3.23 | 0.07 | 5 | 20 |
|  | SPQ total score | | 20 | 21.45 | 13.49 | 0.31 | 0 | 72 |
|  | WEMBS total score | | 50 | 48.63 | 9.42 | 0.22 | 16 | 70 |
|  | ICU total score | | 20 | 20.87 | 7.88 | 0.18 | 1 | 54 |
|  | BIS total score | | 61 | 61.78 | 9.91 | 0.23 | 34 | 102 |
|  |  |  |  |  |  |  |  |  |
|  | First component score | | 0.33 | 0 | 2.39 | 0.06 | -9.17 | 5.33 |
| Childhood family experiences | APQ positive | | 11 | 10.79 | 2.82 | 0.06 | 3 | 15 |
|  | APQ inconsistent | | 6 | 6.39 | 2.53 | 0.06 | 3 | 15 |
|  | APQ poorsup | | 6 | 6.52 | 2.58 | 0.06 | 3 | 15 |
|  | APQ involve | | 10 | 9.35 | 2.66 | 0.06 | 3 | 15 |
|  | APQ punish | | 3 | 3.72 | 1.59 | 0.04 | 3 | 15 |
|  | MOPS maternal abuse | | 0 | 0.89 | 1.86 | 0.04 | 0 | 15 |
|  | MOPS maternal indiff | | 0 | 1.08 | 2.26 | 0.05 | 0 | 18 |
|  | MOPS maternal control | | 3 | 3.15 | 2.55 | 0.06 | 0 | 12 |
|  | MOPS paternal abuse | | 0 | 1.29 | 2.63 | 0.06 | 0 | 15 |
|  | MOPS paternal indiff | | 0 | 2.08 | 3.91 | 0.09 | 0 | 18 |
|  | MOPS paternal control | | 2 | 2.34 | 2.4 | 0.06 | 0 | 12 |
|  |  |  |  |  |  |  |  |  |
|  | First component score | | -0.48 | 0 | 2.03 | 0.05 | -3.16 | 12.32 |
|  |  |  |  |  |  |  |  |  |

## Table S2, Results principle component analyses for PSF and Childhood family experiences

|  |  |  |  |  |
| --- | --- | --- | --- | --- |
| N=1890 | Psychosocial functioning (PSF) |  | Childhood family experiences |  |
| First component |  |  |  |  |
| SE | 2.39 |  |  | 2.03 |
| %var | 0.44 |  |  | 0.37 |
| Factor loadings: |  |  |  |  |
| MFQ | -0.37 |  | APQ positive parenting | 0.33 |
| RCMAS | -0.36 |  | APQ inconsistent parenting | -0.13 |
| LOI | -0.29 |  | APQ poor supervision | -0.24 |
| BEHTOT | -0.24 |  | APQ involvement | 0.33 |
| K10 | -0.35 |  | APQ corporal punishment | -0.23 |
| APSD | -0.25 |  | MOPS maternal abuse | -0.35 |
| CADS_Prosoc | 0.12 |  | MOPS maternal indifference | -0.34 |
| CADS_NegEmo | -0.29 |  | MOPS maternal control | -0.33 |
| CADS_daring | -0.01 |  | MOPS paternal abuse | -0.34 |
| SPQ | -0.33 |  | MOPS paternal indifference | -0.33 |
| WEMBS | 0.31 |  | MOPS paternal control | -0.30 |
| ICU | -0.23 |  |  |  |
| BIS | -0.24 |  |  |  |
|  |  |  |  |  |

## Regressions between relationship childhood family experiences and PSF

The relationship between childhood family experiences (CFE) on PSF. This relationship could be described as quadratic (Figure 1) (Est=-0.76, SE=0.03, t= -24.87, P<2e-16 & ^2: Est=0.05, SE=0.006, t=7.32, P=3.66e-13). This quadratic model fitted the data better (F(1,1887)=53.58, P=3.659e-13) than a linear model (Est=-0.61, SE=0.02, t= -26.45, P<2e-16, R^2^=.27). In this quadratic relationship, childhood family experiences explained 29% of the variance in PSF. A cubic model (Figure S1) (Est=-0.76, SE=0.03, t= -24.82, P<2e-16 & ^2: Est=0.08, SE=0.014, t=5.97, P= 2.88e-09, ^3: Est=-0.004, SE=0.001, t=-2.94, P=.003), fitted the data slightly better than the quadratic model (F1,1886)=8.61, p=.003). However, the cubic model similarly explained 29% of variance, and there was only a marginal improvement when using Bayesian information criteria (BIC quadratic: 8043.466, BIC cubic: 8042.398). Therefore, for reasons of parsimony, we retained the quadratic model instead. Note that the cubic model revealed virtually identical results (see Table S3).

## Table S3 predictors of resilient functioning as calculated from the cubic relationship between PSF and childhood family experiences.

|  |  |  |  |  |  |  |  |
| --- | --- | --- | --- | --- | --- | --- | --- |
| Baseline |  | Est | beta | SE | t | Pr(>\|t\|) |  |
| (N=1890) | Friendship | 0.20 | 0.41 | 0.01 | 17.78 | < 2e-16 | *** |
|  | Family | 0.02 | 0.07 | 0.01 | 2.85 | 0.00 | ** |
|  | Age | 0.05 | 0.07 | 0.01 | 3.30 | 0.00 | *** |
|  | Gender | 0.33 | 0.08 | 0.08 | 3.94 | 0.00 | *** |
|  | SES | 0.00 | -0.01 | 0.00 | -0.41 | 0.69 |  |
|  |  |  |  |  |  |  |  |

## PCA for longitudinal analysis sample (N=1093)

For the longitudinal analyses we re-ran all analyses in the sample that had complete data on all measures at both times (N=1093). At time 1, the first component of a PCA for PSF explained 44% of variance. At time 2, a PCA revealed a first component that explained 46% of variance in PSF (see Table S5 for the loadings). Next we re-ran the PCA for childhood family experiences. To reduce the impact of the timing of the assessment on recall of childhood family experiences (Shaffer *et al.* 2008) we added both time points in one PCA. The resulting first component in this PCA explained 32% variance in the APQ and MOPS assessments at time 1 and 2 (Table S5). We then predicted PSF (time 1 *or* 2) from childhood family experiences in two separate regression analyses. Both models showed that childhood family experiences were a predictor of PSF of comparable magnitudes. At time 1 we found a quadratic relation between childhood family experiences and PSF (Est=-0.51, SE=.03, t=-16.56, p<2e-16, ^2: Est=0.02, SE=.005, t=4.16, p=3.38e-05, R^2^adj=.24). This relation fit the data better than a linear relationship (F(1,1090)=17.34, P=.3.376e-05). A cubic relationship was not better than the quadratic relationship (F(1,1089)=0.07, p=.79). At time 2, a similar quadratic relationship was established between childhood family experiences and PSF (Est=-0.56, SE=.03, t=-17.68, p<2e-16,^2: Est=0.03, SE=.006, t=4.55, p=5.96e-06, R^2^adj=.27). This model fits the data better than a linear model (F(1,1090)=20.71, p=5.957e-06), whereas a cubic model did not improve the model (F(1,1089)=.39, P=.53). From these regressions, we extracted individual residual scores at times 1 and 2 as a proxy for degree of resilient functioning.

## Table S4, First components for PSF and childhood family experiences in N=1093.

|  |  |  |  |  |  |  |
| --- | --- | --- | --- | --- | --- | --- |
| N=1093 | Psychosocial functioning | |  | Childhood family experiences | | |
| First component | T1 | T2 |  |  | T1&T2 | |
| SE | 2.40 | 2.45 |  |  | 2.66 |  |
| %var | 0.44 | 0.46 |  |  | 0.32 |  |
| Factor loadings: | T1 | T2 |  |  | T1 | T2 |
| MFQ | -0.37 | -0.36 |  | APQ positive parenting | 0.25 | 0.25 |
| RCMAS | -0.36 | -0.36 |  | APQ inconsistent parenting | -0.08 | -0.08 |
| LOI | -0.28 | -0.27 |  | APQ poor supervision | -0.16 | -0.15 |
| BEHTOT | -0.23 | -0.23 |  | APQ involvement | 0.25 | 0.24 |
| K10 | -0.35 | -0.35 |  | APQ corporal punishment | -0.16 | -0.16 |
| APSD | -0.25 | -0.24 |  | MOPS maternal abuse | -0.25 | -0.25 |
| CADS_Prosoc | 0.13 | 0.16 |  | MOPS maternal indifference | -0.25 | -0.25 |
| CADS_NegEmo | -0.29 | -0.27 |  | MOPS maternal control | -0.22 | -0.22 |
| CADS_daring | 0.01 | 0.01 |  | MOPS paternal abuse | -0.24 | -0.23 |
| SPQ | -0.33 | -0.33 |  | MOPS paternal indifference | -0.23 | -0.23 |
| WEMBS | 0.32 | 0.32 |  | MOPS paternal control | -0.21 | -0.20 |
| ICU | -0.24 | -0.25 |  |  |  |  |
| BIS | -0.24 | -0.24 |  |  |  |  |
|  |  |  |  |  |  |  |

## Figure S1. The Cubic relationship between PSF and Childhood family experiences


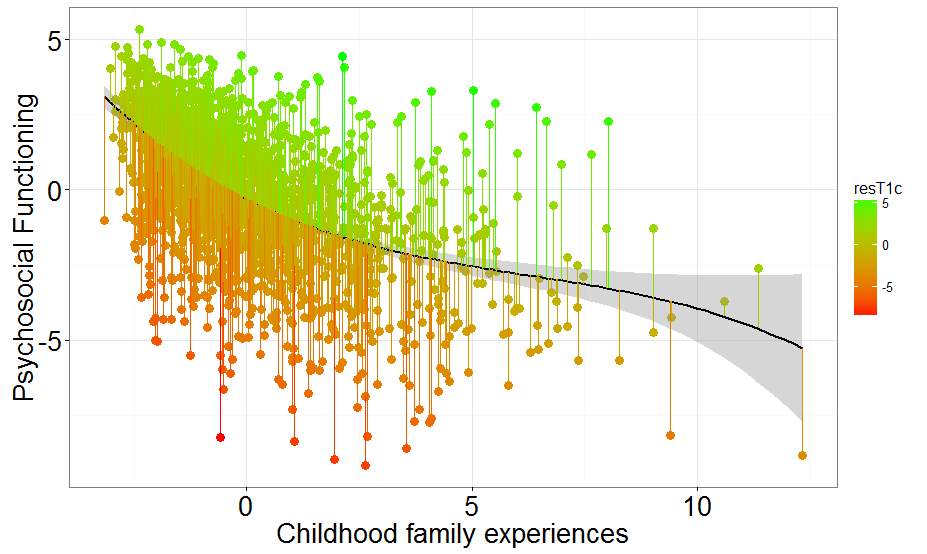


## Neuroscience in Psychiatry Network (NSPN) Consortium author list

*Chief investigator*: Ian Goodyer

*Principal investigators:* Edward Bullmore, Raymond Dolan, Peter Fonagy, Peter Jones,

*Associated faculty:* Peter Dayan, Paul Fletcher, John Suckling, Nikolaus Weiskopf, Pasco Fearon

*Project managers:* Becky Inkster, Gita Prabhu

*Postdoctoral research associates and associated research fellows:* Eran Eldar, Tobias Hauser, Konstantinos Ioannidis, Rogier Kievit, Gemma Lewis, Alda Mita, Michael Moutoussis, Sharon Neufeld, Ela Polek-MacDaeid, Rafael Romero-Garcia, Lizanne Schweren, Michelle St Clair, Jan Stochl, Roger Tait, Beatrice Kiddle, Umar Toseeb, Anne-Laura van Harmelen, Petra Vértes, Kirstie Whitaker, Geert-Jan Will, Gabriel Ziegler,Jorge Zimbron

*PhD & MSc students:* Joost Haarsma, Sian Emma Davies, Juliet Griffin, Michael Hart, Jakob Seidlitz, Max Shinn, František Váša, Konrad Wagstyl, Jessica Fritz

*Data managers:* Cinly Ooi, Barry Widmer

*Research assistants:* Ayesha Alrumaithi, Sarah Birt, Kalia Cleridou, Hina Dadabhoy, Ashlyn Firkins, Sian Granville, Elizabeth Harding, Alexandra Hopkins, Daniel Isaacs, Janchai King, Clare Knight, Danae Kokorikou, Christina Maurice, Cleo McIntosh, Jessica Memarzia, Harriet Mills, Ciara O’Donnell, Sara Pantaleone, Jennifer Scott, Alison Stribling, Nicole Creasy

*Administration team:* Junaid Bhatti, Neil Hubbard, Natalia Ilicheva, Michael Kentell, Ben Wallis, Laura Villis
